# Physiological and gene transcription assays to assess responses of mussels to environmental changes

**DOI:** 10.7717/peerj.7800

**Published:** 2019-10-04

**Authors:** Katrina L. Counihan, Lizabeth Bowen, Brenda Ballachey, Heather Coletti, Tuula Hollmen, Benjamin Pister, Tammy L. Wilson

**Affiliations:** 1 Alaska SeaLife Center, Seward, AK, United States of America; 2 US Geological Survey, Western Ecological Research Center, Davis, CA, United States of America; 3 US Geological Survey, Alaska Science Center, Anchorage, AK, United States of America; 4 Inventory and Monitoring Program, Southwest Alaska Network, National Park Service, Anchorage, AK, United States of America; 5 College of Fisheries and Ocean Sciences, University of Alaska—Fairbanks and Alaska SeaLife Center, Seward, AK, United States of America; 6 Ocean Alaska Science and Learning Center, National Park Service, Anchorage, AK, United States of America; 7 Department of Natural Resource Management, South Dakota State University, Brookings, SD, United States of America

**Keywords:** *Mytilus trossulus*, Gene transcription, Biomarker, Nearshore marine ecosystem, Alaska, Ecosystem management, Monitoring

## Abstract

Coastal regions worldwide face increasing management concerns due to natural and anthropogenic forces that have the potential to significantly degrade nearshore marine resources. The goal of our study was to develop and test a monitoring strategy for nearshore marine ecosystems in remote areas that are not readily accessible for sampling. Mussel species have been used extensively to assess ecosystem vulnerability to multiple, interacting stressors. We sampled bay mussels (*Mytilus trossulus*) in 2015 and 2016 from six intertidal sites in Lake Clark and Katmai National Parks and Preserves, in south-central Alaska. Reference ranges for physiological assays and gene transcription were determined for use in future assessment efforts. Both techniques identified differences among sites, suggesting influences of both large-scale and local environmental factors and underscoring the value of this combined approach to ecosystem health monitoring.

## Introduction

As a result of rapid changes to the global climate and increases in anthropogenic stressors, challenges are mounting along coastal regions around the world. These pressures have the potential to significantly degrade nearshore marine resources and are a threat not only in developed areas, but also in remote regions, including coastal areas of Alaska (Breitburg & Riedel, 2005). Alaska has tens of thousands of miles of coastline that are only accessible by boat or plane and during short periods of the summer due to remoteness and weather conditions. Recognizing that change is inevitable, technologies for monitoring the status of nearshore marine ecosystems in remote areas are necessary for managing and maintaining healthy coastal communities. One approach for assessing ecosystem status is the use of indicator species, including intertidal bivalves (Beyer et al., 2017).

Intertidal bivalve communities are spatially and temporally variable (Bodkin et al., 2017) and are vulnerable to a wide range of influences and disturbances (Denny & Wethey, 2001; Menge & Branch, 2001). Species composition, abundance, and physiology are influenced by a complex variety of stressors, the cumulative effects of which are generally not well understood (Gunderson, Armstrong & Stillman, 2016). In Alaska, nearshore communities have been affected by earthquakes, including the major 1964 Alaska earthquake (Baxter, 1971), volcanic eruptions (DeGange et al., 2010; Walker et al., 2013; Jewett & Drew, 2014; Zimmermann et al., 2018), and hydrocarbon contamination from the 1989 *Exxon Valdez* oil spill (Armstrong et al., 1995; Driskell et al., 1996; Lees, Houghton & Driskell, 1996; Fukuyama, Shigenaka & Hoff, 2000). Further stressors will be forthcoming with increases in ocean temperature, decreases in pH (Lesser et al., 2010; Hoegh-Guldberg & Bruno, 2010; Gaylord et al., 2011; Bijma et al., 2013), contaminants (Franzellitti et al., 2010), and human harvest of marine species (Jamieson, 1993).

Intertidal invertebrates are important members of nearshore communities, and in the Gulf of Alaska are a primary food source for a variety of marine and terrestrial vertebrate and invertebrate predators including brown bears (Smith & Partridge, 2004), sea stars (Paul & Feder, 1975; Fukuyama & Oliver, 1985), shorebirds (Gill Jr & Handel, 1990), sea ducks (Lewis, Esler & Boyd, 2007), sea otters (Calkins, 1978; Doroff & DeGange, 1994; Coletti et al., 2016) and human subsistence users (Fall & Field, 1996). Bay mussels (*Mytilus trossulus*) are ubiquitous throughout nearshore communities in the Gulf of Alaska and northeast Pacific. Changes in bay mussel populations due to abiotic or biotic factors may result in alterations of the entire intertidal community structure (Harley, 2011; Navarrete, Menge & Daley, 2000). For example, a reduction in mussel abundance due to predation or thermal stress decreased overall species diversity in rocky intertidal communities (Harley, 2011).

Nearshore species can serve as a focal point for understanding variables that influence the nearshore ecosystem, as they integrate marine and terrestrial stressors into their behavior and condition and reflect these drivers in their abundance and population trends over time. In our work, bay mussels were selected as an indicator species because they are abundant, an important component of the nearshore food web and, as prodigious filter feeders, will consume and sequester contaminants. Studies using mussels have included assessment of contaminants and changing climate conditions (Akaishi et al., 2007; Beyer et al., 2017; Coray, St.Jean & Bard, 2007; Gagné et al., 2007; Halldórsson, Svavarsson & Granmo, 2005; Halldórsson et al., 2008; Shaw et al., 2011), which are frequent goals of long-term monitoring.

In recent years, there have been major advances in assays to evaluate the physiological condition of mussels using biomarkers (biological metrics that quantify a physiological response including detection of proteins or their activity) and gene transcription (measurement of alterations in transcription of specific genes) to elucidate changes at the molecular level in response to environmental changes and contaminant exposure (Livingstone et al., 2000; Evans & Hofmann, 2012; Hüning et al., 2013; Bolognesi & Cirillo, 2014). Gene transcription is the process by which information from the DNA template of a particular gene is transcribed into messenger RNA (mRNA) and eventually translated into a functional protein. The amount of a particular gene that is expressed is physiologically dictated by a number of intrinsic and extrinsic factors, and analysis of mRNA and protein levels can provide information about dynamic changes in the functional state of an organism. A holistic approach that combines biomarker and gene transcription is gaining momentum in other regions, with researchers evaluating and applying these methods to mussels and other invertebrates for environmental monitoring to improve sensitivity and efficiency (Banni et al., 2017; Beyer et al., 2017; Carella et al., 2018; Sforzini et al., 2018). An advantage of measuring gene transcription is that mRNA production is the earliest observable sign an organism is responding to a stressor, but mRNA is generally not as stable as proteins (De Sousa Abreu et al., 2009; McLoughlin et al., 2006). While protein levels respond more slowly than mRNA, their often greater stability makes assessing protein biomarkers advantageous. Thus, combining the two approaches can be complementary as increases in gene transcripts and proteins are often detected concurrently, but they can also provide information that may be missed when using one method alone.

Often, large scale investigations into populations and ecosystems have been driven by catastrophic changes such as oil spills (Wells, Butler & Hughes, 1995; Peterson et al., 2003), mortality events (Lessios, 1988) and ecological extinction (Jackson et al., 2001). However, investigations ‘after the fact’ may be constrained by a lack of baseline data, limiting insight into ecological pathways and causes of change and recovery (Breitburg & Riedel, 2005). The goal of this study was to work toward an approach that combines biomarker and genetic methods to assess the condition of nearshore ecosystems, including those in areas that are remote and logistically difficult to access. The specific objectives to accomplish this goal included: (1) acquire baseline physiological and gene transcription data for the bay mussel across sites in the Gulf of Alaska, (2) assess the relationship between physiological and gene transcription assays for validation and support of both techniques, and (3) determine if site-level differences were present. Mussels were sampled in 2015 and 2016 from six intertidal sites in two national parks. The sites were expected to reflect a range of representative habitat types to generate reference baseline data applicable to mussels in this region. We hypothesized that we would be able to establish baseline data for the sites that were sampled and that the biomarker and gene transcription assays would provide complementary results. Further, we hypothesized that biomarker and gene transcription results would not be significantly different among sites.

## Materials & Methods

### Study organisms and area

**Figure 1:**
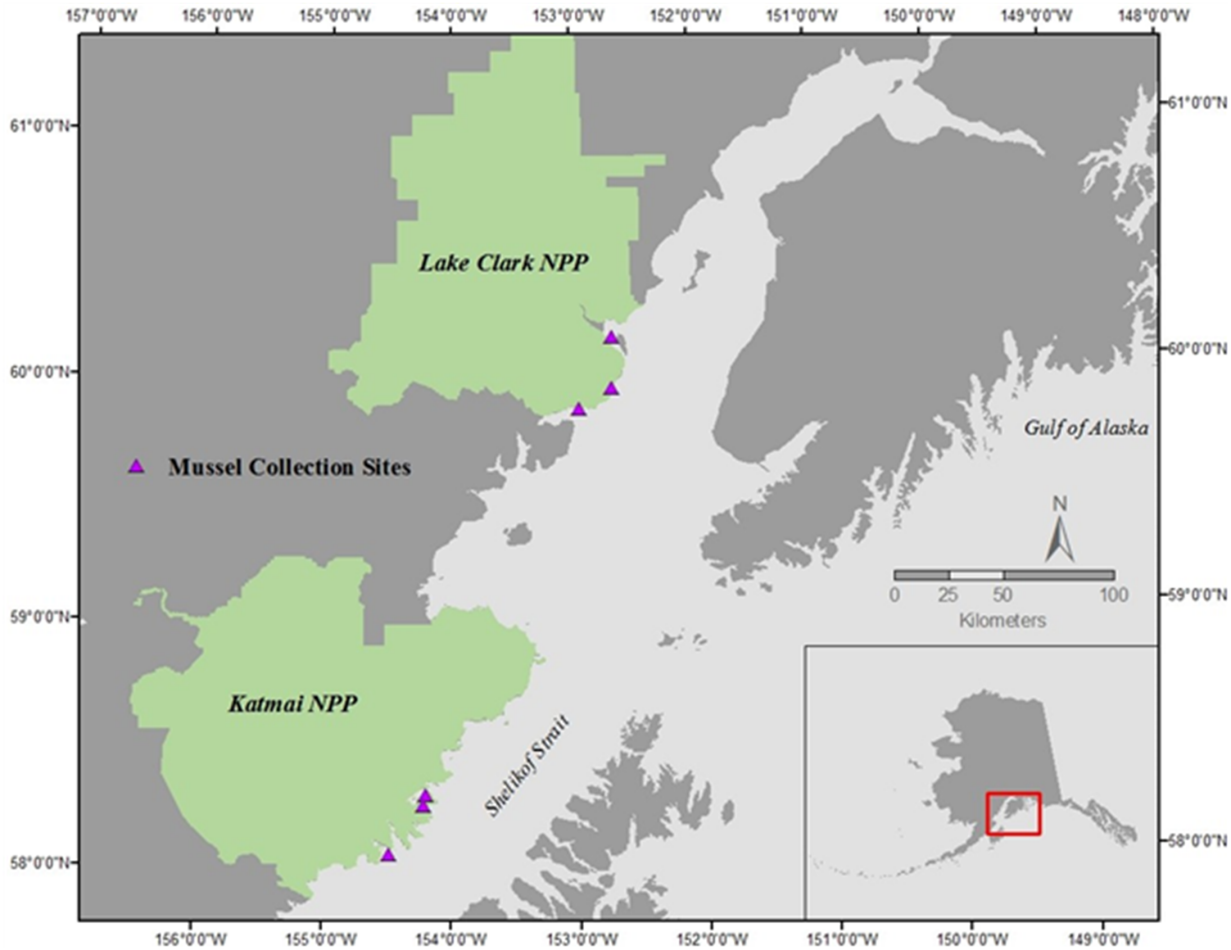
Mussel sampling sites in Lake Clark National Park and Preserve (NPP) and Katmai National Park and Preserve (NPP). Sites were sampled during June of 2015 and 2016.

Mussels (*Mytilus trossulus*) were collected during late morning on the rising tide in July of 2015 and 2016 at three sites within each of two national parks in southcentral Alaska: Lake Clark National Park and Preserve (LACL; sites: Fossil Point, Silver Salmon, and Chinitna Bay) and Katmai National Park and Preserve (KATM; sites: Kukak, Kaflia, & Takli) (Fig. 1) (Alaska Department of Fish and Game permit # CF-15-088 for KATM and LACL, 2015, and # CF-16-089 for KATM and LACL, 2016). Due to the remote nature of these parks, all sites are only accessible by boat or plane during the summer months. Twenty mussels were collected from each site in both 2015 and 2016 (total of 120 each year), with 10 designated for physiological assays and 10 for gene transcription; we therefore collected a total of 240 mussels, and used 120 of them for each methodology. Mussels collected in KATM were from sites that were randomly selected as part of a long-term nearshore monitoring program (Dean, Bodkin & Coletti, 2014). These sites were established on mixed-sediment beaches in protected to semi-protected areas (Weitzman et al., 2017). Currently, no long-term monitoring of coastal bivalve resources exists along the LACL coastline, so sampling locations in LACL were selected based on the presence of mussels. Mussels were present on and collected from bedrock outcroppings. Overall, our intent was to capture the range of natural variation across a set of sites that are representative of coastline habitats in lower Cook Inlet and the Gulf of Alaska.

The mussels were kept submerged in seawater and processed as soon as possible following collection, generally within 1–4 h. For biomarker assays, hemolymph was extracted from the posterior adductor muscle with a tuberculin needle. The hemolymph and mussel were frozen in liquid nitrogen for transport to the lab where they were transferred to a −80 °C freezer until processing.

For gene transcription assays, gill tissue was removed, placed in cryovials with RNAlater^®^, and frozen at −20 °C until analyses.

### Invertebrate physiology

Seven biomarkers were used to assess the physiological status of the mussels and are summarized in Table 1.

**Table 1:** Biomarkers selected for this study and their primary functions and interactions.

**Biomarker**	**Biological Process**	**Environmental Interaction**	**References**
Condition factor	Growth	Ocean acidification Nutrient availability Contaminants Temperature	Carmichael, Shriver & Valiela (2004); Gagné et al. (2007)
Shell Thickness	Growth	Ocean acidification Nutrient availability Predation Density	Blanchard & Feder (2000); Freeman (2007); Gaylord et al. (2011); Xavier, Branch & Wieters (2007)
Hemocyte Count	Immune function	Contaminants Pathogens	Galloway & Depledge (2001)
Hydrogen Peroxide	Immune function	Contaminants Pathogens	Galloway & Depledge (2001)
RNA:DNA	Metabolic condition	Nutrient availability	Caldarone et al. (2001), Lesser et al. (2010)
Cytochrome P450	Detoxification	Contaminants	Rewitz et al. (2006)
Heat Shock Protein 40	Thermal stress	Temperature Stress	Cruz-Rodriguez & Chu (2002)

### Morphometrics, condition factor and age

The length, width, height, and total wet weight of mussels were measured prior to dissection. The posterior adductor muscle and digestive gland were excised for assays. The condition factor was calculated by dividing the total mussel weight by the shell length. Age was determined by counting shell growth rings. The median and range of morphometrics and ages by site are summarized in Table 2.

**Table 2:** Medians and ranges of morphometrics and ages of mussels collected at six sites in Lake Clark and Katmai National Parks and Preserves during 2015 and 2016 (*n* = 10 mussels per site per year).

			Length (mm)		Width (mm)		Height (mm)		Weight (g)		Age (years)	
Park	Site	Year	Median	Range	Median	Range	Median	Range	Median	Range	Median	Range
Katmai	Takli	2015	41.81	22.27-56.28	21.63	19.98–25.81	16.93	15.05–20.74	6.15	4.48–11.34	6	5–10
		2016	41.72	33.36–44.92	19.29	17.09–22.83	16.01	13.47–18.70	5.91	3.99–9.19	7	5–9
	Kukak	2015	38.73	29.91–48.79	19.75	15.90–24.03	15.51	12.03–18.74	4.08	1.68–7.78	6.5	4–8
		2016	34.69	30.76–44.22	18.06	16.18–21.01	13.56	11.32–16.26	4.05	2.24–7.36	7	6–11
	Kaflia	2015	48.94	44.20–57.96	23.39	21.17–26.36	19.47	17.09–22.82	8.11	6.56–13.91	9	7–12
		2016	37.24	30.67–45.80	17.12	15.97–19.87	14.11	12.16–17.45	4.28	2.11–7.65	7	5–10
Lake Clark	Fossil Point	2015	60.26	41.14–78.55	25.94	20.23–32.73	25.06	16.03–30.06	16.18	4.57–24.19	8	5–11
		2016	54.73	42.11–63.74	24.99	23.13–29.44	22.51	18.66–25.90	10.53	5.90–19.12	9	6–12
	Silver Salmon	2015	48.02	37.14–53.83	22.54	19.16–25.74	22.36	15.94–23.80	9.06	4.16–12.72	7	5–12
		2016	45.88	41.96–55.39	21.46	20.02–26.19	18.55	14.51–24.95	6.12	4.73–14.89	8	5–12
	Chinitna Bay	2015	42.76	37.73–57.16	20.48	17.06–23.85	20.44	16.34–25.45	9.02	5.32–14.68	7	5–8
		2016	41.69	33.61–46.19	20.07	15.43–22.45	20.23	14.87–24.31	9.43	4.65–14.05	7.5	6–12

### Shell thickness

A micrometer was used to measure the shell thickness at 5 regularly spaced points around the shell approximately one mm from the edge (Versteegh & Hansson, 2012).

### Hemocyte count

A 0.01 ml sample of hemolymph was diluted 1:1 with tris-buffered saline (TBS) and the number of cells were counted using a hemocytometer in three replicates (Akaishi et al., 2007).

### Hydrogen peroxide production

Hemolymph samples were diluted 1:1 with tris-buffered saline (TBS). Samples were tested in triplicate by pipetting 0.05 ml of the 1:1 hemolymph:TBS solution into a 96-well plate. After incubating the plate in the dark for 1 h, 0.05 ml of phenol red solution (phosphate buffered saline pH 7.4, 5.5 mM dextrose, 0.56 mM phenol red, 8.5 U ml^−1^ horseradish peroxidase, type II) was added to each well and incubated for another 30 min in the dark. The reaction was stopped by adding 0.01 ml of 1 N NaOH, and the plate was read on a Molecular Devices SpectraMax Plus microplate reader (Sunnyvale, CA, USA) at 620 nm (Akaishi et al., 2007).

### RNA:DNA ratio

Half of the posterior adductor muscle was homogenized and 0.15 ml of 1% sarcosyl tris-EDTA (STEB) was added to the homogenate. The mixture was vortexed for 60 min and then 1.35 ml of tris-EDTA (TE) buffer was added. The sample was centrifuged 15 min at 14,000 x g at room temperature and the supernatant saved for testing. The samples were diluted 1:20 and 0.075 ml of each sample was added to a 96-well plate in duplicate. Genomic, unsheared DNA from calf thymus (Sigma-Aldrich, St. Louis, MO) was used to prepare a DNA standard curve (0.1 µg ml^−1^, 0.2 µg ml^−1^, 0.4 µg ml^−1^, 0.8 µg ml^−1^, 1.6 µg ml^−1^, 3.2 µg ml^−1^, 6.4 µg ml^−1^, 10.0 µg ml^−1^). RNA from bovine pancreas (Sigma-Aldrich) was used for a RNA standard curve (0.4 µg ml^−1^, 0.8 µg ml^−1^, 1.6 µg ml^−1^, 3.0 µg ml^−1^, 6.0 µg ml^−1^, 8.0 µg ml^−1^, 12.0 µg ml^−1^, 16.0 µg ml^−1^). The wells had 0.075 ml of ethidium bromide solution (2 µg ml^−1^) added to them and the microplate was shaken for 15 min. The plate was read in a SpectraMax Gemini EM fluorescent microplate reader (Molecular Devices) with 525 nm excitation, 600 nm emission to determine the total nucleic acid reading. Each well had 0.0075 ml of RNase solution (20 U ml^−1^) added to it and the plate was shaken for 20 min and read on the microplate reader again with the same settings. The second reading was the DNA only reading. The RNA content was determined by subtracting the second reading from the first (Caldarone et al., 2001).

### Cytochrome P450 activity

The digestive gland was homogenized in buffer (25 mM Hepes, 125 mM NaCl, 0.1 mM EDTA, 0.1 mM dithiothreitol) at a 1:5 weight:volume ratio. The mixture was centrifuged at 1,500 × g for 10 min at 2 °C and the supernatant transferred to a clean tube. The supernatant was centrifuged at 10,000 × g for 20 min at 2 °C. The supernatant was discarded and the pellet resuspended in 0.15 ml of microsome buffer (25 mM Hepes, 140 mM NaCl, 1 mM KH_2_PO_4_). A 96-well plate was inoculated with 0.05 ml of each microsome sample in triplicate. Each well had 0.05 ml of 50 µM BFC solution (7-benzyl-4(trifluoromethyl) coumarin in phenol red-free Dulbecco’s Modified Eagle Medium) added and the plate was incubated for 4 h at room temperature. The plate had 0.04 ml of stop solution (80% CH_3_CN, 20% 0.5 M Tris base) added to each well and it was read on a SpectraMax Gemini EM fluorescent microplate reader (Molecular Devices) at 410 excitation, 530 emission (Mensah-Osman et al., 2007).

### Heat shock protein

Half of the posterior adductor muscle was homogenized and lysed in buffer (150 mM NaCl, 1% Triton-X 100, 0.5% sodium deoxycholate, 0.1% sodium dodecyl sulphate, 50 mM Tris, 1 mM phenylmethylsulfonyl fluoride). The homogenate was centrifuged at 12,000 rpm for 20 min at 4 °C and the supernatant collected. The protein concentration was determined using a Bradford assay. The sample was mixed 1:1 with 2×Laemmli buffer, boiled at 100 °C for 5 min and loaded on a SDS-PAGE gel. Each gel was run with a positive heat shock protein control and a molecular weight marker. The gel was electrophoretically transferred onto a polyvinyl difluoride (PVDF) membrane. Total protein was visualized by staining the gel with Pierce reversible protein stain (Thermo Scientific, Pittsburgh, PA USA). The stain was removed and the membrane was probed with a mouse anti-HSP 40 primary antibody (Abcam, Cambridge, MA USA). The membrane was washed and incubated with a secondary alkaline phosphatase labeled anti-mouse antibody (Abcam). The membrane was washed again and alkaline phosphatase substrate added. The membrane was photographed to document bands and analyzed with Image Studio Lite software, version 5.2.5 (Li-Core, Lincoln, NE, USA).

### Invertebrate gene transcription

Transcription of 14 genes was used to assess the physiologic status of the mussels; genes used are summarized in Table 3.

**Table 3:** Genes selected for the transcription panel and their primary functions and interactions.

**Gene**	**Biological process**	**Environmental interaction**	**References**
Calmodulin (CaM)	Metabolism, shell formation	Ocean acidification Temperature Dissolved oxygen	Chen et al. (2012); Li et al. (2004)
Caspase 8 (Casp 8)	Apoptosis, necrosis, inflammation	Pathogens Contaminants	Romero et al. (2011)
Macrophage migration inhibitory factor (MIF)	Innate immunity	Pathogens	Parisi et al. (2012), Philipp et al. (2012)
Calponin (CNN)	Hypoxia	Ocean acidification Dissolved oxygen	Hüning et al. (2013), Li et al. (2016)
Chitinase (CHI)	Metabolism, hypoxia	Ocean acidification Dissolved oxygen	Banni et al. (2011), Hüning et al. (2013)
Cytochrome C Oxidase IV (CCOIV)	Hypoxia	Dissolved oxygen	Fukuda et al. (2007)
Heat shock protein 70 (HSP70)	Thermal stress, molecular chaperone	Temperature Pathogens Contaminants Hypoxia	De Maio (1999), Iwama et al. (1999), Tsan & Gao (2004)
Heat hock protein 90 (HSP90)	Thermal stress, molecular chaperone	Temperature Pathogens Contaminants Hypoxia	De Maio (1999), Iwama et al. (1999), Tsan & Gao (2004)
Hypoxia-inducible factor alpha (HIFa)	Hypoxia	Dissolved oxygen	Wu (2002)
Myticin B (MytB)	Innate immunity	Pathogens	Balseiro et al. (2011)
Mytilin (Myt)	Innate immunity	Pathogens Ocean acidification	Balseiro et al. (2011), Mitta et al. (2000)
Metallothionein 20 (MT20)	Detoxification	Contaminants—metals	Banni et al. (2007)
Cytochrome P450, family 3 (Cyp3)	Detoxification	Contaminants	Giuliani et al. (2013)
Tumor protein 53 (p53)	Apoptosis	Contaminants—PAHs	Goodson et al. (2006), Banni et al. (2009)

Stability of the proposed reference gene (18S) was determined using the web-based analysis tool RefFinder (http://www.leonxie.com/referencegene.php) (Xie et al., 2012). Sufficient stability was found, thus cycle threshold crossing values (C_T_) for the genes of interest were normalized to 18S. Although sex can potentially have impacts on gene transcription, most studies investigating gene transcription in the specific genes identified in our panel do not differentiate between sexes (Zeng et al., 2012; Woo et al., 2011; Lacroix et al., 2014; Balseiro et al., 2011; Hüning et al., 2013; Woo et al., 2013; Cellura et al., 2007; Châtel et al., 2015; Franzellitti et al., 2010; Place, O’Donnell & Hofmann, 2008; Li et al., 2010; Giannetto et al., 2015; Dondero et al., 2006; Sureda et al., 2011; Zanette et al., 2013; Giuliani et al., 2013; Di et al., 2011), and we did not differentiate by sex in this study.

### Tissue collection and RNA extraction

Gill tissue was collected from each mussel and placed immediately into RNAlater^®^ (Ambion/Life Technologies, Grand Island, New York). All tissue samples were stored at −20 °C. Total RNA was extracted from pulverized gill tissue using the RNeasy Lipid Tissue Mini Kit (Qiagen; http://www.qiagen.com). To remove contaminating genomic (g)DNA, the spin columns were treated with 10 U µl ^−1^ of RNase-free DNase I (DNase, Amersham Pharmacia Biotech Inc.; http://www.apbiotech.com) at 20 °C for 15 min. RNA was then stored at −80 °C pending further analyses.

### cDNA synthesis

A standard cDNA synthesis was performed on 2 µg of RNA template from each mussel. Reaction conditions included 4 units reverse transcriptase (Omniscript, Qiagen, Valencia, CA, USA), 1 µM random hexamers, 0.5 mM each dNTP, and 10 units RNase inhibitor, in RT buffer (Qiagen, Valencia, CA, USA). Reactions were incubated for 60 min at 37 °C, followed by an enzyme inactivation step of 5 min at 93 °C, and then stored at −30 °C until further analysis.

### Primer design

Degenerate primers were designed based upon multi-species alignments (GenBank). Briefly, degenerate primer pairs developed for the mussel were used on cDNA from three randomly selected mussel samples. Degenerate primer pairs were designed to amplify 14 genes of interest and one ribosomal housekeeping gene (Table 4). The PCR amplifications using these primers were performed on 20 ng of each cDNA sample in 50 µl volumes containing 20–60 pmol of each primer, 40 mM Tris-KOH (pH 8.3), 15 mM KOAc, 3.5 mM Mg (OAc)_2_, 3.75 µg/ml bovine serum albumin (BSA), 0.005% Tween-20, 0.005% Nonidet-P40, 200 µM each dNTP, and 5U of Advantage^®^ 2 Taq polymerase (Clontech, Palo Alto, CA, USA). The PCR was performed on an MJ Research PTC-200 thermal cycler (MJ Research, Watertown, MA, USA) and consisted of 1 cycle at 94 °C for 3 min, and then 40 cycles at 94 °C for 30 s, at 60 ° C for 30 s, and 72 °C for 2 min, with a final extension step of 72 °C for 10 min. The products of these reactions were electrophoresed on 1.5% agarose gels and resulting bands visualized by ethidium bromide staining. Definitive bands representing PCR products of a predicted base pair size of the targeted gene were excised from the gel, and extracted and purified using a commercially available nucleic acid-binding resin (Qiaex II Gel extraction kit; Qiagen, Valencia, CA, USA).

**Table 4:** *Mytilus trossulus*-specific quantitative real-time polymerase chain reaction primers used in the analysis of mussels.

**Gene**	**Forward primer sequence (5′–>3′)**	**Reverse primer sequence (5′–>3′)**
*CaM*	TCTGTTCGACAAAGATGGCG	GCATCTACTTCGTTAATCATGT
*Casp 8*	CCCAACCAGTAGTAACACCAGAC	GTATGAACCATGCCCCTATATCA
*MIF*	TACACCCAGACCAAATGATG	TTCTCCTAATGCTCCAATACTG
*CNN*	ATACTCCGGCGGAGACAGT	TCTTCTTCGGGAATCTCTTGT
*CHI*	ATATCATCTACTCATTCGCCA	AGTGATAGTTTCAAGGCTG
*CCOIV*	GATGTAGTGGCTCTCAAGGAT	AGATCTGTTTCCATTCACCTGT
*HSP70*	GGTGGTGAAGACTTTGACAACAG	CTAGTTTGGCATCACGTAGAGC
*HSP90*	GATCTCCAACTCATCTGATGC	GTGTGTTGTTATCCTTGTCTG
*HIFa*	ATACCTTGGCATCTCACAGAT	GACTTCTTCTTGTTGGTGGTC
*MytB*	AATGTCTTCGTTGTTCCAG	AATGCCAGTTTCACCTTG
*Myt*	GTTATTCTGGCTATCGCTCTTG	GTATAATGTCAAACAGAACGGGTC
*MT20*	GATCTACTAAGCAGACCAGC	TACATCCGGAACATCCACAG
*Cyp3*	AGTTACAGTACTTGGACAGATTCGT	TGCCTCAAGTAATGCCAGCCTCA
*P53* *18s*	TGTGTAGACTGAGGGATTCATTGG GTGCTCTTGACTGAGTGTCTCG	TCACCTTCTTCATCAGTTTGTTTTT CGAGGTCCTATTCCATTATTCC

### Real-time PCR

Real-time PCR reactions for the individual genes of interest and the housekeeping gene (18S) were run in separate wells (Bowen et al., 2018). Briefly, 1 µl of cDNA was added to a mix containing 12.5 µl of QuantiTect Fast SYBR Green^®^ Master Mix (5 mM Mg 2 +) (Qiagen, Valencia, CA, USA), 0.5 µl each of forward and reverse sequence specific primers (Invitrogen, Carlsbad, CA, USA), and 10.5 µl of RNase-free water; total reaction mixture was 25 µl. The reaction mixture cDNA samples for each gene of interest and 18S were loaded into Fast 96 well plates in duplicate and sealed with optical sealing tape (Applied Biosystems, Foster City, CA, USA). Reaction mixtures that contained water but no cDNA were used as negative controls.

### Statistical analysis

Analysis of qPCR data was conducted using normalized C _T_ values (housekeeping gene threshold crossing subtracted from the gene of interest threshold crossing); the lower the normalized value, the more transcripts are present. A change in normalized value of 2 is approximately equivalent to a 4-fold change in the amount of the transcript. A normalized value of 34 or higher indicated a quantity less than the detection limit for transcription of that gene. Two samples contained values that were less than the detection limit for transcription and were thus omitted from statistical analyses because they were influential outliers that affected within and among site means.

For all data, gene transcription and biomarker assays, medians, 2.5% and 97.5% percentiles, and ranges were calculated (NCSS, Statistical and Power Analysis Software). As mussel age (based on ring counts) and size were not found to have significant effects on biomarkers or gene transcription, they were omitted from further analyses. We used generalized linear mixed effects models (GLMM) to estimate site means for each gene transcription factor and biomarker parameter. We included sampling year as a random effect to account for variances introduced by collecting sample units over the course of 2 years. Input data were examined for normality prior to analysis; a log linear model was used for hemocyte count, RNA:DNA ratio, P450, and HSP40 biomarkers to normalize variances, and reported means were back-transformed into the original data scale. We fit separate models for each gene and biomarker with maximum likelihood estimation using the lme4 package in R 3.5.0 (R Development Core Team, 2012), which accounts for the unbalanced data using Satterthwaite’s method. We conducted post-hoc Tukey tests of site level differences. To obtain site means for each year, we fit a Gaussian generalized linear model (GLM) with a site by year interaction. The resulting site means were used to test for correlations between genes and biomarker parameters. Relationships between gene transcript and biomarker data, as well as within gene transcript and biomarker data, were assessed in R using Pearson correlations. We considered correlations that were >0.30 (or <−0.30) to be of possible biological significance. To further evaluate relationships between and within responses, we used R to conduct principal components analysis (PCA). As for the Pearson correlations, we evaluated the relationships within gene transcript and biomarker datasets using all available data records, and the relationships between gene transcripts and biomarkers using site by year means obtained from the GLM interaction model. We visualized multivariate interactions by plotting the contributions of each variable to PC1 and PC2, and by plotting the quality of representation of each metric to all principal components that could be fit. We showed data from all dimensions that could be fit to better represent the exploratory nature of our analysis.

## Results

Biomarker and gene transcript medians, 2.5% and 97.5% percentiles, and ranges were determined for use in future monitoring efforts (Table 5). Site-specific medians and ranges for the biomarker and gene transcription assays for each year are provided as Files S1 and S2.

**Table 5:** Medians, 2.5 and 97.5 percentiles and ranges obtained from 120 mussels collected at 6 sites in Lake Clark and Katmai National Parks and Preserves for all variables across all sites and years. For gene transcription, higher numbers indicate less transcription, and a value of 34 indicates a quantity less than detection limits.

**Condition Factor**			**Shell Thickness (mm)**			**Hemocyte Count(cells mL**^−1^)		
Median	2.5–97.5%	Range	Median	2.5–97.5%	Range	Median	2.5–97.5%	Range
0.16	0.07–0.31	0.06–0.35	0.67	0.27–1.69	0.24–2.22	241,667	13,333–1,540,000	3,333–1,740,000
**Hydrogen Peroxide (OD**_**620**_**)**			**RNA:DNA (ratiog**^−1^**)**			**P450 (activity mg**^−1^**protein)**		
Median	2.5–97.5%	Range	Median	2.5–97.5%	Range	Median	2.5–97.5%	Range
0.05	0.01–0.10	0.01–0.14	69.49	4.09–663.70	1.74–833.49	27.64	9.48–127.94	7.62–190.33
**HSP40(Arbitrary Units)**			**CaM (C**_**T**_**)**			**Casp8 (C**_**T**_**)**		
Median	2.5–97.5%	Range	Median	2.5–97.5%	Range	Median	2.5–97.5%	Range
2.88	0.32–24.98	0.18–40.03	16.19	13.91–19.42	13.57–21.46	10.48	8.26–12.91	7.74–13.82
**MIF (C**_**T**_**)**			**CNN (C**_**T**_**)**			**CHI (C**_**T**_**)**		
Median	2.5–97.5%	Range	Median	2.5–97.5%	Range	Median	2.5–97.5%	Range
17.88	12.37–22.17	11.92–23.22	25.12	21.67–27.93	17.70–34	18.66	16.01–23.97	13.30–34
**CCOIV (C**_**T**_**)**			**HSP70 (C**_**T**_**)**			**HSP90 (C**_**T**_**)**		
Median	2.5–97.5%	Range	Median	2.5–97.5%	Range	Median	2.5–97.5%	Range
18.38	12.73–22.29	11.56–23.14	11.31	7.99–14.65	6.63–16.58	13.54	9.66–16.66	9.31–17.89
**HIFa (C**_**T**_**)**			**MytB (C**_**T**_**)**			**Myt (C**_**T**_**)**		
Median	2.5–97.5%	Range	Median	2.5–97.5%	Range	Median	2.5–97.5%	Range
14.13	12.24–16.43	11.86–17.02	12.6	5.80–18.69	4.67–19.72	15.91	12.02–19.24	10.69–22.11
**MT20 (C**_**T**_**)**			**Cyp3 (C**_**T**_**)**			**P53 (C**_**T**_**)**		
Median	2.5–97.5%	Range	Median	2.5–97.5%	Range	Median	2.5–97.5%	Range
10.14	5.48–15.22	4.43–20.73	14.67	12.01–17.18	11.11–18.10	12.79	10.31–15.77	9.39–34

The Principal Components Analysis for the biomarkers, gene transcripts, and mix of biomarkers and gene transcripts revealed that three, five, and six principal components had eigenvalues >1, respectively. The first three dimensions of the biomarker PCA explained 65% of the variance in the data, and 27% was attributed to the first principal component. The first five dimensions of the gene transcript PCA explained 69% of the variation in the data, with 31% going to the first component. Two-thirds of the variation was explained by six dimensions for the combined PCA, with 33% explained by the first principal. Only 12 dimensions could be fit for the combined dataset because of the sample size restrictions caused by using site means for each year.

**Figure 2:**
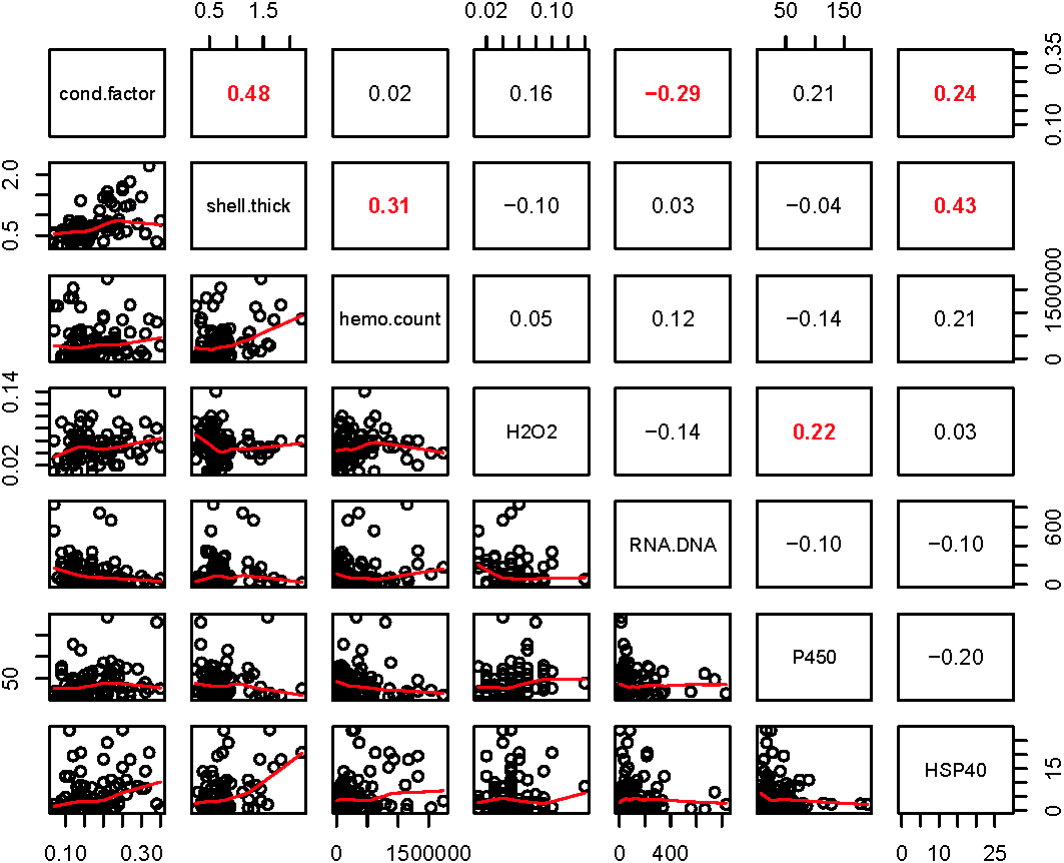
Pearson correlations between biomarkers obtained from 120 mussels collected at six sites in Lake Clark and Katmai National Parks and Preserves. The numbers are Pearson correlations between biomarkers. Bold red numbers are statistically significant correlations (*P* < 0.05). The graphs depict actual observations (black circles) and the best fit line (red) between paired biomarkers.

**Figure 3:**
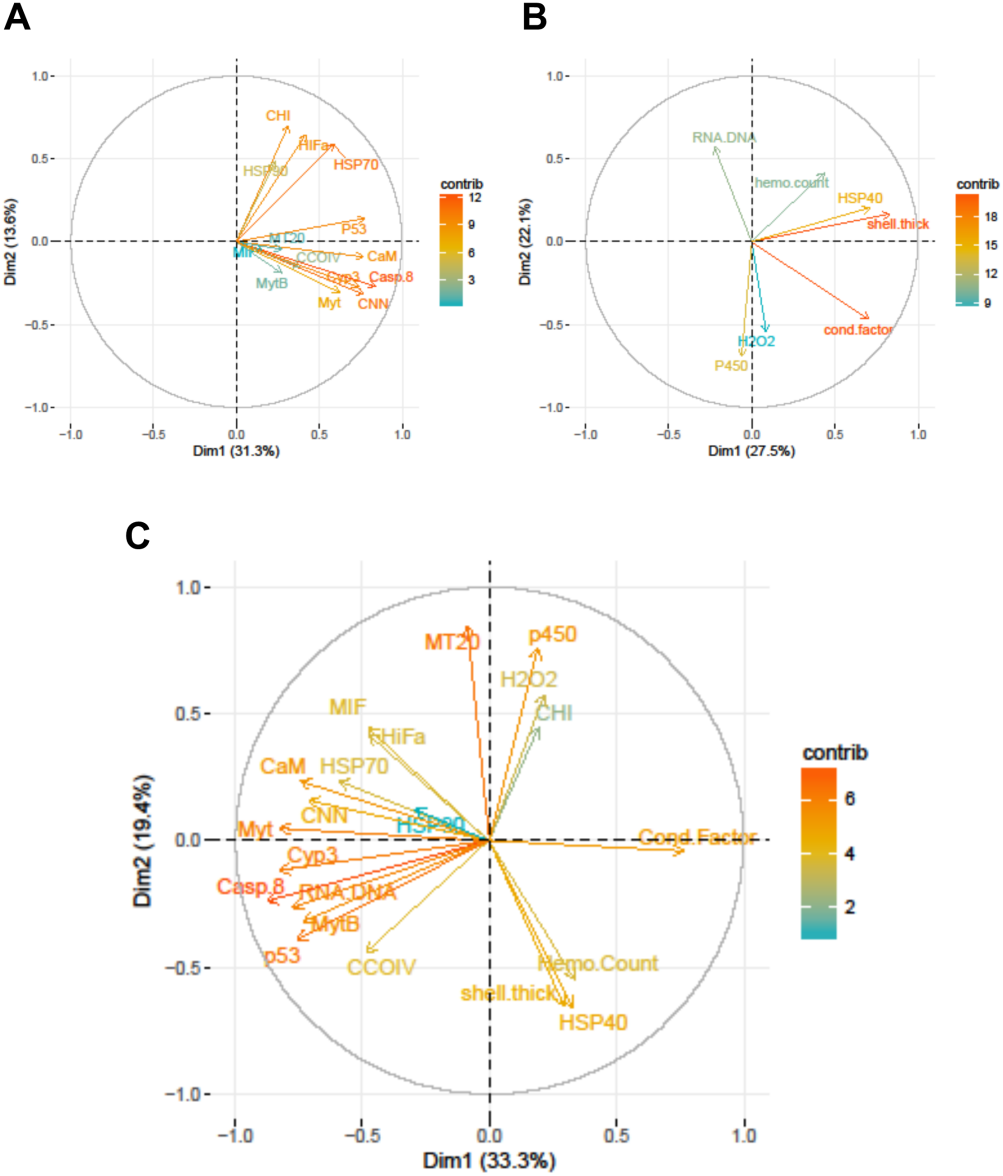
Correlation plots based on Principle Components Analysis of variables measured from 120 mussels collected at six sites in Lake Clark and Katmai National Parks and Preserves. (A) Gene transcripts, (B) biomarkers, (C) all metrics. The arrows correspond to each measured variable and maps the contribution of that variable to the first and second principle components (Dim 1 and 2). Arrows that are close together are correlated, and those that are opposite are anti-correlated. Those orthogonal to one another are not correlated. Variables that contribute strongly to either the first or second axis are nearer the axes than variables that do not contribute strongly. The length and color of the arrow illustrates the strength of the contribution of each variable, with the longer arrows rendered in warm colors contributing more than those that are shorter and rendered in cool colors.

**Figure 4:**
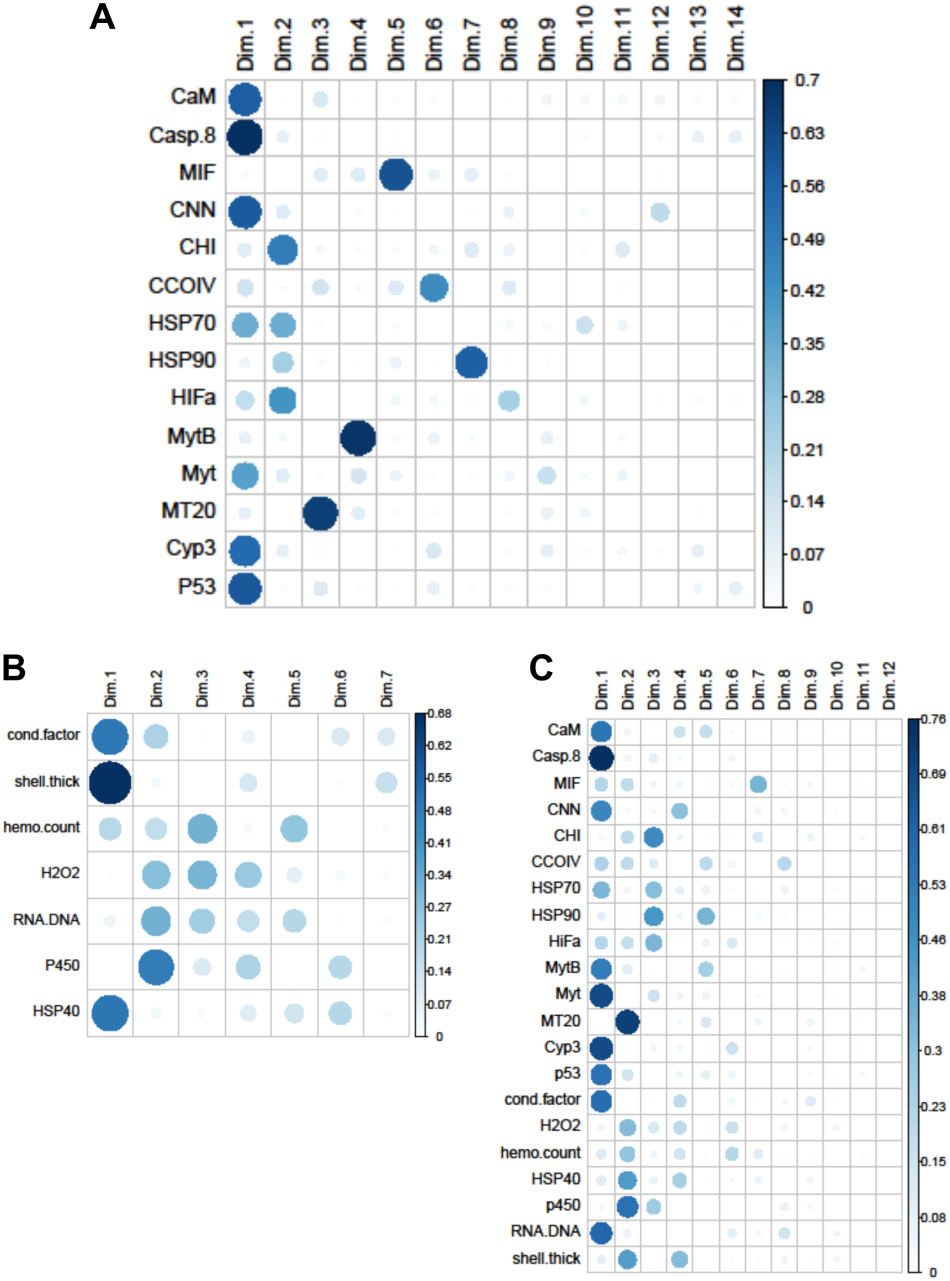
Quality of representation plots based on Principle Components Analyses of variables measured from 120 mussels collected at six sites in Lake Clark and Katmai National Parks and Preserves. (A) gene transcripts, (B) biomarkers, (C) all metrics. The charts depict all metrics and all Principle Components that could be calculated for each analysis. The size and shade of the circle denotes how well the variable is represented by each principle component. The large and dark circles denote good representation, while the small and light circles denote poor representation. This plot has the advantage of showing potential variable correlations in multiple dimensions.

Within the biomarker assays we found correlations between condition factor and shell thickness (Rpearson = 0.48), and condition factor and HSP40 (Rpearson = 0.24, Fig. 2). Condition factor, shell thickness and HSP40 also contributed the most to PC1 when the physiological markers were analyzed by PCA (Fig. 3), with shell thickness having the highest quality of representation on this axis (Fig. 4). Hydrogen peroxide production, P450 activity and RNA:DNA contributed to PC2, with RNA:DNA representing an opposite effect from the other two variables (Fig. 3). PC3 corresponded to hemocyte count. PC1-3 contributed 27.5%, 22.1% and 14.5%, respectively, to variation in the physiological data and 64.1% of overall variation.

**Figure 5:**
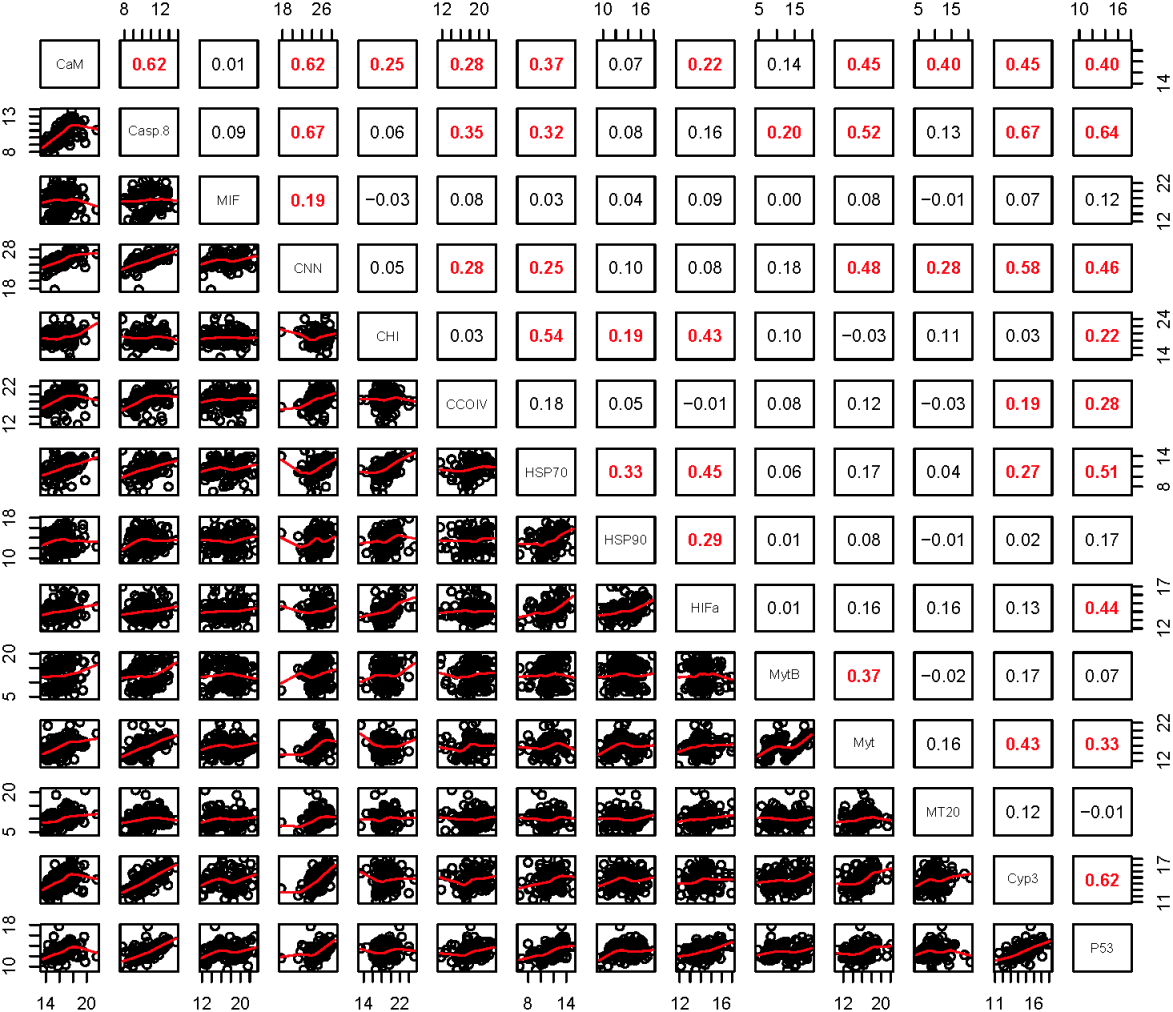
Pearson correlations between gene transcripts obtained from 120 mussels collected at six sites in Lake Clark and Katmai National Parks and Preserves. The numbers are Pearson correlations between gene transcripts. Red numbers are statistically significant correlations (*P* < 0:05). The graphs depict all observations (black circles) and the best fit line (red) between paired gene transcripts.

Strong correlations were noted between many of the gene transcripts (Fig. 5). No strong negative correlations in levels of transcription were noted among the genes. Analysis of gene transcription by PCA indicated no gene transcripts were anticorrelated, matching the Pearson correlation results. PC1 corresponded to CaM, Casp8, CNN, HSP70, Myt, Cyp3 and p53 (Fig. 3) with Casp8 providing the highest quality of representation on this axis (Fig. 4). This was similar to the inference obtained by the Pearson correlations where PC1 maps to variables that have strong correlations (>0.30) with both CaM and Casp8. CHI, HSP70, HSP90 and HIFa contributed the most to PC2 (Fig. 3). MT20 contributed most substantially to PC3. PC1-3 corresponded to 31.3%, 13.6% and 8.9%, respectively, of variation in gene transcription data and 53.8% of variation overall.

Increased P450 activity was associated with increased transcription of 4 genes (most notably CCOIV) and decreased transcription of MT20. A higher condition factor was associated with increased gene transcription: nine of 14 genes had a negative correlation with condition factor that was >0.30. In contrast, we observed an inverse relation between RNA:DNA and gene transcription, with nine of 14 genes having a correlation >−0.30 (Table 6).

**Table 6:** Results of the Pearson correlations between gene transcripts and biomarkers obtained from 120 mussels collected at six sites in Lake Clark and Katmai National Parks and Preserves. Statistically significant correlations (*P* < 0.05) are bolded. Values reflect the numerical correlation; however, the biological correlation will be the inverse, as lower C_T_ values reflect higher levels of gene transcription.

	Condition factor	P450 activity	RNA:DNA
CaM	−0.40	0.04	**0.59**
Casp8	−0.49	−0.18	**0.83**
MIF	−0.33	0.26	0.11
CNN	−0.21	0.04	0.45
CHI	0.22	0.06	−0.25
CCOIV	−0.24	**−0.71**	0.39
HSP70	−0.47	−0.20	0.46
HSP90	−0.16	−0.34	0.18
HiFa	−0.54	−0.05	0.16
Myt B	**−0.60**	−0.34	0.57
Myt	−0.49	0.10	**0.65**
MT20	−0.08	**0.62**	−0.09
Cyp3	**−0.71**	−0.12	0.52

PCA analysis showed that PC1 was comprised primarily of the gene transcripts: CaM, Casp8, CNN, HSP70, MytB, Myt, Cyp3, p53, and the biomarkers RNA:DNA and condition factor, with condition factor having an opposite relationship than the other measures (Fig. 3). PC2 corresponded to MIF, MT20, hydrogen peroxide production, P450, shell thickness and hemocyte count, with shell thickness and hemocyte count opposing the other variables (Fig. 3). CHI, HSP70, HSP90 and HIFa, with Myt, hydrogen peroxide production and P450 activity opposing those measures, contributed the most to PC3. PC1-3 corresponded to 33.3%, 19.4% and 12.5%, respectively, of the variance in the metrics and 65.3% of variance overall.

## Discussion

The overall goal of our research was to use biomarker and genetic methods to assess the condition of nearshore species in remote areas of Alaska, as nearshore ecosystems, particularly at higher latitudes, are vulnerable to changing environmental conditions (Taylor et al., 2017). Our approach included establishment of reference ranges for biomarker and gene transcription assays of mussels collected at sites in LACL and KATM and identification of correlations between the assays. Due to the remote nature of the parks and other logistical constraints, sampling opportunities were limited to a one week window in July 2015 and again in July 2016. Other environmental monitoring studies generally have been conducted in areas with known contamination concerns, allowing for a clear contrast among experimental groups (Akaishi et al., 2007; Banni et al., 2017; Carella et al., 2018; Gagné et al., 2007; Halldórsson et al., 2008; Sforzini et al., 2018; Shaw et al., 2011). However, our goal was to develop a method to monitor sites for changes, rather than assess sites already impacted. Therefore, our collection sites were not known to be compromised by anthropogenic activity and were considered relatively pristine. Any differences in biomarker or gene transcription results were expected to be due to natural variation and to provide a range of representative values which could act as a reference for continuing studies.

In this light, although differences in physiological patterns among sites are of interest, the more important goal is to identify the range of values that can be expected under what we assume to be “normal” conditions. Biomarker and gene transcript levels may differ between two sites, but both sites, nevertheless, may fall within a range considered to be normal. Many factors can cause natural variation in biomarker and gene transcription levels, including predators (Reimer & Tedengren, 1996), temperature, dissolved oxygen (Abele & Puntarulo, 2004) and wave exposure (Dahlhoff & Menge, 1996). There may have been differences in these and other physical (e.g., substrate type) or biological factors across the sites that contributed to normal background variation.

Several considerations must be taken into account when interpreting gene transcription data. First, within an individual, there are tradeoffs in the allocation of limited resources among competing physiological functions (Vera-Massieu et al., 2015). Increased physiological activity and gene transcription in response to stressors can present a metabolic challenge for any species, including bivalves (Graham et al., 2010), and may result in reduction of fitness evidenced by decreased reproductive capability, increased susceptibility to disease, or disadvantageous behavioral changes (Graham et al., 2010; Martin et al., 2010). Additionally, biological processes identified by our panel are influenced by multiple genes, and each gene may contribute to multiple functions. For example, although several genes on our panel respond to ocean acidification, including calmodulin (CaM), calponin (CNN), and chitinase (CHI), each plays a different role in the overall mechanism (Li et al., 2004; Banni et al., 2011; Chen et al., 2012; Hüning et al., 2013; Li et al., 2016). Thus, transcript levels of genes with similar endpoint functions may not necessarily correlate with each other. Identifying patterns of variation for each gene measured (among and within sites) is an important step in determining their value for monitoring studies.

**Figure 6:**
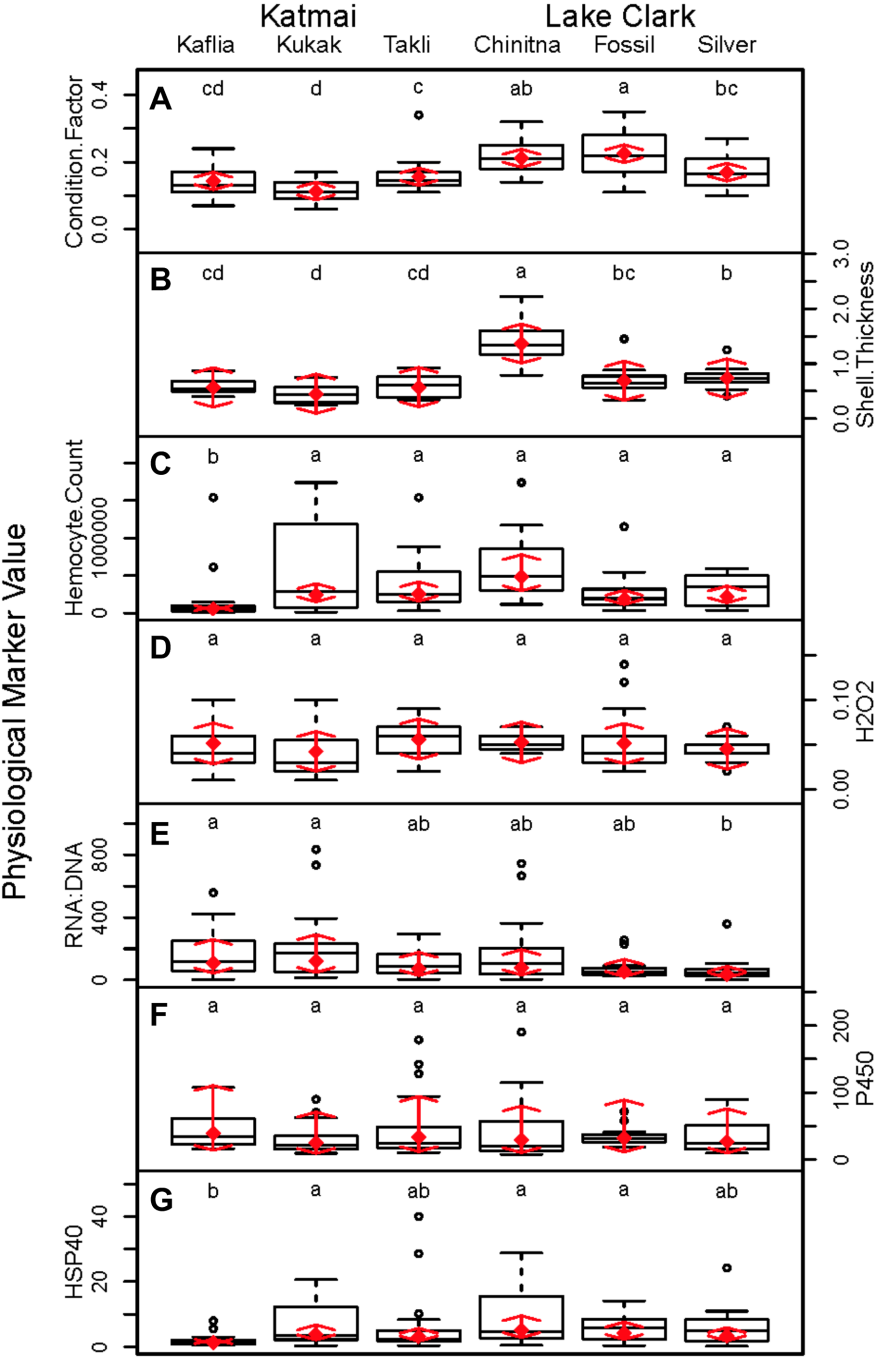
Boxplots of biomarker data obtained from 120 mussels collected at six sites in Lake Clark and Katmai National Parks and Preserves. Random effects model results are denoted by red diamonds (mean) and red arrows (95% confidence intervals). Sites sharing a lowercase letter did not differ statistically based on post-hoc testing (*P* < 0.05). (A) condition factor, (B) shell thickness, (C) hemocyte count, (D) hydrogen peroxide, (E) RNA:DNA, (F) P450 activity, (G) HSP40.

**Figure 7:**
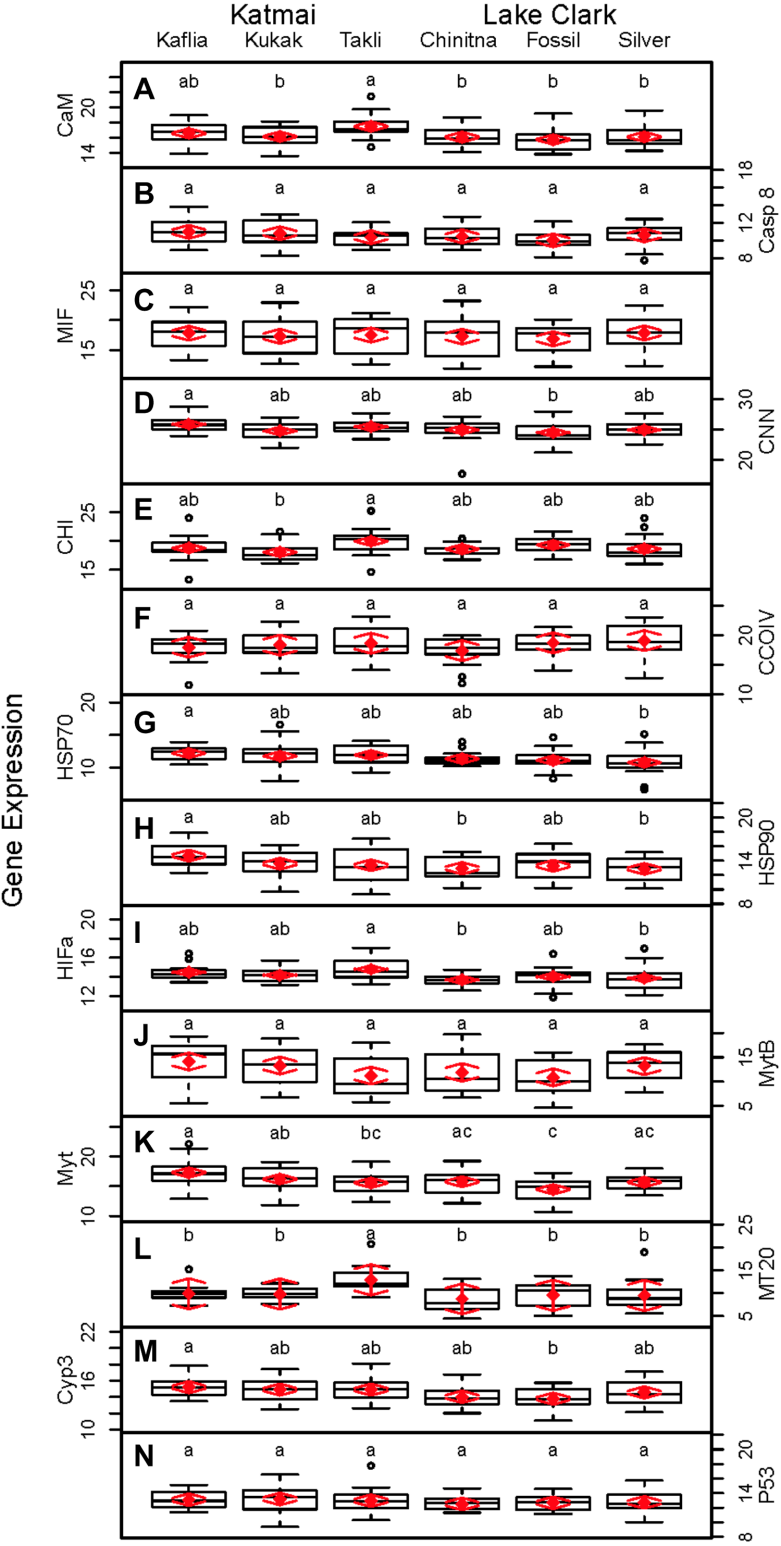
Boxplots of gene transcription data (normalized C_T_ values) obtained from 120 mussels collected at six sites in Lake Clark and Katmai National Parks and Preserves. Random effects model results are denoted by red diamonds (mean) and red arrows (95% confidence intervals). Sites sharing a lowercase letter did not differ statistically based on post-hoc Tukey testing (*P* < 0.05). (A) CaM, (B) Casp8, (C) MIF, (D) CNN, (E) CHI, (F) CCOIV, (G) HSP70, (H) HSP90, (I) HIFa, (J) MytB, (K) Myt, (L) MT20, (M) Cyp3, (N) p53.

Significant differences were observed among sites for biomarker as well as gene transcription parameters. For example, five out of seven biomarkers varied significantly among sites (condition factor, shell thickness, RNA:DNA ratio, HSP40, hemocyte count) (Fig. 6), and nine of 14 genes varied significantly among sites (CNN, CaM, CHI, Cyp3, HIFa, HSP70, HSP90, MT20, and Myt) (Fig. 7). Given that we had no reason to expect differences among sites due to obvious impairments, we might assume that the differences we detected were part of the natural variation we would observe in these populations when sites are compared across a large spatial scale.

Condition factor and shell thickness were higher at Chinitna Bay, Fossil Point, and Silver Salmon (all in LACL) as compared to Kaflia, Kukak, and Takli (all in KATM). Elevated mussel condition factor has been associated with the presence of higher quality and/or quantity of nutrients (Carmichael, Shriver & Valiela, 2004), suggesting that nutrient availability varied between parks. Water originating from Upper Cook Inlet flows along the LACL coastline, eventually merging with the Alaska Coastal Current (Nagorski et al., 2008). The KATM coastline is dominated by the Alaska Coastal Current, which carries a high amount of freshwater to the region (Nagorski et al., 2007). Differences in oceanographic processes between LACL and KATM likely influence nutrient availability along the coast.

Shell thickness can be influenced by changes in predation pressure, mussel density or abiotic factors. Studies conducted with *M. galloprovincialis* have demonstrated that mussels living at higher densities are smaller with thicker shells (Xavier, Branch & Wieters, 2007). Predation can induce mussels to thicken their shells as a defense mechanism (Freeman, 2007). Abiotic factors such as temperature, salinity and wave action can influence shell thickness, as well (Akester & Martel, 2000; Blanchard & Feder, 2000). Based on observations, Chinitna Bay is more exposed than the other sites and mussels at that location had the thickest shells, potentially as a result of experiencing more wave action. However, mussel density, predators and other abiotic factors were not quantified during this study.

Mussel hemocyte count was the most variable biomarker within sites, and other studies have observed similar variability (Akaishi et al., 2007; Coray, St.Jean & Bard, 2007; Duchemin et al., 2008). Mussels at Kaflia had a significantly lower hemocyte count than mussels at the other sites. This result indicates that despite relatively high variability, differences in hemocyte count can be identified. Wild mussels are constantly exposed to antigens that may stimulate an immune response and elevate hemocyte count with high variability between individuals (Galloway & Depledge, 2001). Less variability was observed in the hydrogen peroxide assay suggesting it may be a more suitable biomarker for monitoring immune activity than hemocyte count.

Variability among sites was also observed in RNA:DNA and HSP40. Mussels at Kaflia and Kukak had significantly higher RNA:DNA compared to those from Silver Salmon, indicating differences in protein production among sites. HSP40 levels were higher at Kukak, Chinitna Bay and Fossil Point as compared to Kaflia suggesting an elevated response to an unknown stressor at those three sites.

Relationships within the biomarker assays, within the gene transcription panel, and between the biomarkers and genes were determined using Pearson correlations and PCA, and the results of both analyses were complementary. A positive Pearson correlation was found between condition factor and shell thickness and between shell thickness and HSP40. Additionally, in the PCA analysis, condition factor, shell thickness and HSP40 contributed to PC1 with high quality of representation on that axis. Condition factor indicates the nutritional status of the mussels, and it was not surprising that shell thickness would relate to overall condition as mussels with more nutrients might be expected to allocate more nutrients to shell formation. Mussels with nutrient deficits have been shown to metabolize their shell (Masthanamma, Purushotham & Ramamurthi, 1984). Shell thickness influences body temperature (Caddy-Retalic, Benkendorff & Fairweather, 2011), and possibly mussels with thicker shells absorb more heat, resulting in HSP40 production. Additional research is needed to understand the association between shell formation and HSP40 production.

Given the multiple functions of many of the genes in our transcript panel, as well as the interconnectedness of genes in general, we expected and found numerous correlations among the genes (Fig. 5). For example, CaM, CNN, and CHI are all sensitive to changes in ocean acidification, and Casp8, MIF, MytB, Myt, HSP70, and HSP90 can all be influenced by pathogen exposure. Contaminant exposure can influence the transcription of Casp8, HSP70, HSP90, MT20, Cyp3, and p53, and changes in dissolved oxygen can alter the transcription of CaM, CNN, CHI, CCOIV, and HIFa. As expected, there was a Pearson correlation between CNN and CaM, but not with CHI, as we would have anticipated. The strong correlations observed among CNN, Casp8 and Myt suggest potential links among processes associated with shell formation and pathogen presence. In addition, Casp8 was strongly correlated with Cyp3, MytB, and p53, suggesting a link between pathogen and contaminant exposures. CaM, Casp8, CNN, HSP70, Myt, Cyp3 and p53 contributed substantially to PC1 according to the PCA.

An objective of our study was the comparison of the two methodologies, gene transcription and biomarker assays. Several of the biomarkers and genes were associated with similar physiological functions, and we anticipated correlations would arise. Although we did not expect complete agreement, we expected the methods to support one another. Several correlations between the biomarker and gene transcription assays were identified. CaM, Casp8, CNN, HSP70, MytB, Myt, Cyp3, p53, RNA:DNA and condition factor contributed to PC1 in the PCA analysis of all metrics, with condition factor having an opposing relationship to the other variables. A negative Pearson correlation between condition factor and MytB, Cyp3 and p53 was identified, while a positive correlation between RNA:DNA and CaM, Casp8, Myt and p53 was present. The statistical analysis used the C_T_ values for gene transcription, and high C_T_ values indicate low gene expression. Therefore, the relationship between high gene expression and high condition factor makes biological sense, as a good condition factor implies ample resources, which translates into an overall ability to increase transcription of genes needed to combat stressors. RNA:DNA was associated with lower transcription of several genes, which was not an expected finding. However, the small number of genes in this study (14) may not be representative of patterns in the overall transcriptome, which numbers many thousands of genes.

Higher activity of P450 was strongly associated with lower MT20 transcription and increased CCOIV transcription in Pearson correlations. In PCA analysis, MT20 and P450 contributed to PC2. MT20 and P450 are responsible for detoxifying metals and xenobiotics, respectively. Contaminants can contain complex chemical mixtures that induce different detoxification pathways (Islam & Tanaka, 2004). CCOIV is transcribed during hypoxic conditions, and contaminants can damage or interfere with gill function inhibiting respiration and resulting in hypoxia (Sokolova & Lannig, 2008). We expected P450 activity to correlate with Cyp3 transcription, but this was not observed. The cytochrome P450 family, which includes Cyp3, contains numerous proteins (Rewitz et al., 2006). The gene transcription assay is specific for Cyp3, but the P450 assay detects the enzyme activity of multiple cytochrome isoforms, not only Cyp3 (Kobayashi et al., 2002). Therefore, overall P450 enzyme activity may be increased while Cyp3 transcription is not.

The results of this study examined the strength of using both gene transcription and biomarker assays to evaluate coastal ecosystems. This approach has been implemented for mussel biomonitoring in other coastal regions including the Mediterranean (Carella et al., 2018; Sforzini et al., 2018). In both of these studies, site-level differences were detected using genetic techniques and biomarker analyses. The results correlated with the presence of known contamination (Carella et al., 2018; Sforzini et al., 2018). In our study, differences between sites also were identified, but without known sources of contamination. A limitation of our findings is that biomarker and gene transcription assays were not run on the same individual mussels (i.e., ten mussels were collected for each set of assays at each site in each year). Therefore, our correlations are computed on a site basis, and we are not able to evaluate how metrics from the two sets of assays relate on an individual mussel basis.

At the beginning of this study, we made assumptions about the pristine nature of the intertidal sites (Weeks, 1999; Weeks, 2003). The sites are along Alaskan national park coastlines, with very little direct anthropogenic stressors. However, variations in biomarkers and genes suggest some differences exist among sites. Most notably, mussels from LACL were in better condition with thicker shells, perhaps associated with quantity and/or quality of nutrients in the vicinity of those sites. Developing reference biomarker and gene transcription levels in mussels is important to properly differentiate among changes due to climate or anthropogenic activity and natural variation. The capability to discern local and large-scale changes is beneficial for monitoring remote locations where sampling opportunities are limited. Effects of some environmental impacts, such as oil spills, may be readily observed, but subtle changes in the marine environment can be difficult to detect. The sensitivity of biomarker and gene transcription assays supports the identification of subtle changes. Additionally, this approach enables the measurement of cumulative effects of several stressors. However, further studies comparing the biomarker and gene transcription assays, under controlled conditions and in the field, with both sets of assays measured on the same individual mussels, are needed to provide a more thorough understanding of how the various metrics respond and relate.

In addition to the need for controlled exposure studies, our results highlight an important reality pertaining to ecosystem monitoring: although combined technologies provide better resolution of potential causative factors, an approach using longitudinal monitoring to continually assess populations for subtle yet significant changes is necessary to provide insight into ecosystem health. In the future, we will improve our understanding of ecosystems at risk if we take a proactive approach to monitoring prior to occurrence of population level effects (Bennett, Thompson & Mortenson, 2006).

## Conclusions

Intertidal communities are important in marine and terrestrial food webs and understanding the condition of these intertidal resources will support management in the maintenance of healthy coastal ecosystems. This study generated baseline gene transcription and biomarker data that will be useful for monitoring these remote areas of the Alaskan coastline for environmental change. The gene transcription and biomarker assays were advantageous because they provided a significant amount of information regarding physiological responses of mussels to environmental conditions from small amounts of tissue, which is necessary for assessing remote intertidal communities where sampling opportunities and environmental data are constrained by logistics. Additionally, the assays were sensitive enough to detect differences between sites with no obvious impacts. Results between the gene transcription and biomarker assays were often complementary, but some differences were noted, likely due to variations in the rate of production and turnover of mRNA and proteins. Integrating gene transcription and biomarker assays provided a more comprehensive assessment of mussel condition than either approach alone. Additional controlled experiments will strengthen our understanding of the value of this approach. We anticipate implementing this holistic approach to evaluate stressors affecting intertidal communities and changes occurring within those communities over time.

##  Supplemental Information

10.7717/peerj.7800/supp-1 File S1 Site-specific medians and ranges for the physiological assays during each year

10.7717/peerj.7800/supp-2 File S2 Site-specific medians and ranges for the gene transcription assays during each year

10.7717/peerj.7800/supp-3 Supplemental Information 1 R code

10.7717/peerj.7800/supp-4 Data S1 Gene transcription data

10.7717/peerj.7800/supp-5 Data S2 Physiological data

## Additional Information and Declarations

### Field Study Permissions

The following information was supplied relating to field study approvals (i.e., approving body and any reference numbers): Field collections were approved by the National Park Service. Field collection permit numbers are Alaska Department of Fish and Game permit # CF-15-088 for Katmai and Lake Clark National Parks and Preserves, 2015, and # CF-16-089 for Katmai and Lake Clark National Parks and Preserves, 2016.

### Data Availability

The following information was supplied regarding data availability: The raw measurements and code used in the statistical analyses are available in the Supplemental Files.
