# Supplementary material for: Physiological and gene transcription assays to assess responses of mussels to environmental changes
Source: PeerJ. 2019 Oct 4;7:e7800. doi: 10.7717/peerj.7800 (PMC6779115; doi:10.7717/peerj.7800)
Supplement: File S1 [file peerj-07-7800-s001.docx]

|  |  |  | Condition Factor | | Shell Thickness (mm) | | | | Hydrogen Peroxide (OD_620_) | | | Hemocyte Count (cells mL^-1^) | | |
| --- | --- | --- | --- | --- | --- | --- | --- | --- | --- | --- | --- | --- | --- | --- |
| Park | Site | Year | Median | Range | Median | | Range | | Median | Range | | Median | | Range |
| Katmai | Takli | 2015 | 0.15 | 0.11-0.34 | 0.39 | | 0.34-0.47 | | 0.07 | 0.05-0.09 | | 388,333 | | 110,000-883,333 |
|  |  | 2016 | 0.14 | 0.11-0.20 | 0.76 | | 0.59-0.92 | | 0.04 | 0.02-0.07 | | 186,667 | | 32,000-1,540,000 |
|  | Kukak | 2015 | 0.11 | 0.06-0.17 | 0.29 | | 0.24-0.38 | | 0.05 | 0.02-0.09 | | 250,667 | | 16,667-1,740,000 |
|  |  | 2016 | 0.11 | 0.07-0.17 | 0.57 | | 0.44-0.75 | | 0.02 | 0.01-0.10 | | 283,333 | | 13,333-1,326,000 |
|  | Kaflia | 2015 | 0.17 | 0.13-0.24 | 0.51 | | 0.40-0.55 | | 0.06 | 0.04-0.10 | | 80,000 | | 43,333-146,667 |
|  |  | 2016 | 0.12 | 0.07-0.17 | 0.66 | | 0.49-0.87 | | 0.03 | 0.01-0.09 | | 31,667 | | 3,333-1,540,000 |
| Lake Clark | Fossil Point | 2015 | 0.27 | 0.11-0.35 | 0.58 | | 0.34-0.88 | | 0.06 | 0.04-0.14 | | 181,667 | | 73,333-1,153,333 |
|  |  | 2016 | 0.20 | 0.14-0.30 | 0.74 | | 0.58-1.45 | | 0.03 | 0.02-0.03 | | 216,667 | | 33,333-364,000 |
|  | Silver Salmon | 2015 | 0.19 | 0.10-0.24 | 0.72 | | 0.41-0.90 | | 0.04 | 0.02-0.07 | | 100,000 | | 36,667-376,667 |
|  |  | 2016 | 0.14 | 0.11-0.27 | 0.75 | | 0.58-1.25 | | 0.05 | 0.04-0.06 | | 498,333 | | 300,000-593,333 |
|  | Chinitna Bay | 2015 | 0.20 | 0.14-0.26 | 1.17 | | 0.79-1.58 | | 0.06 | 0.04-0.07 | | 355,000 | | 116,667-660,000 |
|  |  | 2016 | 0.23 | 0.14-0.32 | 1.60 | | 1.22-2.22 | | 0.05 | 0.04-0.06 | | 855,333 | | 193,333-1,740,000 |
|  |  |  | RNA:DNA g^-1^ | | | P450 (activity mg^-1^ protein) | | | | HSP40 (Arbitrary Units) | | |  |  |
| Park | Site | Year | Median | Range | | Median | | Range | | Median | Range | |  |  |
| Katmai | Takli | 2015 | 48.21 | 4.05-145.39 | | 86.13 | | 17.10-178.65 | | 1.72 | 0.32-4.56 | |  |  |
|  |  | 2016 | 148.00 | 37.73-294.39 | | 18.62 | | 10.07-32.44 | | 5.26 | 2.22-40.03 | |  |  |
|  | Kukak | 2015 | 49.22 | 15.40-106.93 | | 30.56 | | 13.24-89.90 | | 3.07 | 0.28-13.90 | |  |  |
|  |  | 2016 | 230.95 | 171.30-833.49 | | 15.80 | | 9.12-46.34 | | 5.07 | 1.76-20.62 | |  |  |
|  | Kaflia | 2015 | 58.83 | 3.11-337.55 | | 52.54 | | 16.18-107.34 | | 1.55 | 0.51-2.98 | |  |  |
|  |  | 2016 | 203.50 | 92.48-557.92 | | 25.61 | | 15.71-77.47 | | 1.09 | 0.53-7.95 | |  |  |
| Lake Clark | Fossil Point | 2015 | 47.05 | 24.63-61.07 | | 33.90 | | 18.51-71.66 | | 8.50 | 0.34-14.04 | |  |  |
|  |  | 2016 | 77.34 | 25.27-255.02 | | 31.41 | | 20.52-35.04 | | 3.89 | 1.16-9.98 | |  |  |
|  | Silver Salmon | 2015 | 45.11 | 1.74-105.21 | | 50.84 | | 13.26-89.52 | | 5.71 | 0.18-10.90 | |  |  |
|  |  | 2016 | 47.92 | 13.48-358.04 | | 16.41 | | 9.47-35.40 | | 2.21 | 0.31-24.21 | |  |  |
|  | Chinitna Bay | 2015 | 139.04 | 15.58-744-28 | | 61.24 | | 15.71-190.33 | | 2.61 | 0.43-10.91 | |  |  |
|  |  | 2016 | 52.59 | 3.60-266.04 | | 12.93 | | 7.62-26.86 | | 18.15 | 3.39-28.76 | |  |  |
