# Supplementary material for: Physiological and gene transcription assays to assess responses of mussels to environmental changes
Source: PeerJ. 2019 Oct 4;7:e7800. doi: 10.7717/peerj.7800 (PMC6779115; doi:10.7717/peerj.7800)
Supplement: File S2 [file peerj-07-7800-s002.docx]

|  |  |  | Calponin | | Calmodulin | | Casp8 | | CCOIV | |
| --- | --- | --- | --- | --- | --- | --- | --- | --- | --- | --- |
| Park | Site | Year | Median | Range | Median | Range | Median | Range | Median | Range |
| Katmai | Takli | 2015 | 25.15 | 23.40-27.05 | 16.90 | 14.77-19.76 | 10.10 | 8.92-12.06 | 17.79 | 14.09-23.14 |
|  |  | 2016 | 25.31 | 24.26-27.66 | 17.40 | 16.81-21.46 | 10.65 | 9.38-11.59 | 20.51 | 16.73-21.69 |
|  | Kukak | 2015 | 24.24 | 21.97-34.00 | 15.49 | 13.57-16.79 | 9.84 | 8.26-10.61 | 17.14 | 13.55-21.21 |
|  |  | 2016 | 25.79 | 23.51-26.95 | 17.40 | 15.57-18.15 | 12.24 | 10.12-12.95 | 19.55 | 17.23-22.29 |
|  | Kaflia | 2015 | 25.31 | 23.94-26.69 | 15.77 | 13.91-17.40 | 10.19 | 8.91-11.89 | 17.13 | 11.56-19.08 |
|  |  | 2016 | 25.85 | 24.70-28.73 | 17.66 | 16.46-18.98 | 12.10 | 8.92-13.82 | 19.31 | 17.35-20.74 |
| Lake Clark | Fossil Point | 2015 | 25.49 | 23.27-27.93 | 16.37 | 13.83-19.20 | 9.96 | 8.54-12.16 | 19.29 | 14.03-21.39 |
|  |  | 2016 | 23.62 | 21.21-24.52 | 15.38 | 13.99-16.40 | 9.89 | 8.08-10.66 | 18.23 | 17.05-19.98 |
|  | Silver Salmon | 2015 | 24.99 | 22.56-26.06 | 15.48 | 14.88-17.96 | 10.27 | 7.74-11.69 | 17.72 | 16.35-22.45 |
|  |  | 2016 | 24.83 | 23.24-27.62 | 16.15 | 14.28-19.61 | 11.09 | 9.24-12.43 | 19.56 | 12.73-23.04 |
|  | Chinitna Bay | 2015 | 26.18 | 17.70-34.00 | 17.61 | 15.30-34.00 | 11.43 | 9.61-34.00 | 17.81 | 11.82-34.00 |
|  |  | 2016 | 24.64 | 23.58-26.91 | 15.35 | 14.11-16.98 | 9.79 | 8.93-12.69 | 18.08 | 14.99-19.96 |
|  | | | | | | | | | | |
|  |  |  | Chitinase | | Cyp3 | | HIFa | | HSP70 | |
| Park | Site | Year | Median | Range | Median | Range | Median | Range | Median | Range |
| Katmai | Takli | 2015 | 18.74 | 14.64-21.91 | 15.27 | 13.53-18.10 | 14.19 | 13.25-17.02 | 10.91 | 9.25-13.29 |
|  |  | 2016 | 20.75 | 18.60-25.24 | 14.36 | 12.60-16.70 | 15.24 | 13.36-15.83 | 13.36 | 10.86-14.11 |
|  | Kukak | 2015 | 18.74 | 16.11-34.00 | 14.14 | 12.49-16.84 | 14.15 | 13.15-15.71 | 11.91 | 8.13-16.58 |
|  |  | 2016 | 17.16 | 16.49-21.63 | 15.46 | 13.46-17.42 | 14.15 | 13.40-14.56 | 12.18 | 7.99-13.05 |
|  | Kaflia | 2015 | 19.21 | 17.91-24.02 | 14.65 | 13.49-15.83 | 14.01 | 13.60-15.92 | 12.35 | 10.49-13.76 |
|  |  | 2016 | 18.31 | 13.30-19.75 | 15.83 | 13.74-17.82 | 14.35 | 13.43-16.43 | 12.41 | 10.70-13.92 |
| Lake Clark | Fossil Point | 2015 | 19.27 | 16.76-20.34 | 13.51 | 11.11-15.69 | 13.63 | 12.24-16.40 | 11.42 | 8.31-13.34 |
|  |  | 2016 | 19.68 | 17.36-21.64 | 14.05 | 11.50-15.74 | 14.37 | 11.86-14.98 | 11.00 | 8.79-14.65 |
|  | Silver Salmon | 2015 | 17.95 | 16.01-23.97 | 13.76 | 12.13-15.62 | 13.79 | 12.11-16.96 | 10.32 | 6.63-15.13 |
|  |  | 2016 | 18.10 | 17.38-22.42 | 15.83 | 12.44-17.11 | 13.70 | 12.73-15.96 | 10.89 | 9.44-13.83 |
|  | Chinitna Bay | 2015 | 19.30 | 17.90-34.00 | 14.06 | 12.79-34.00 | 13.81 | 12.58-34.00 | 11.41 | 10.40-34.00 |
|  |  | 2016 | 18.12 | 16.74-20.44 | 13.85 | 12.01-16.76 | 13.74 | 13.26-14.37 | 11.24 | 10.20-12.15 |
|  | | | | | | | | | | |
|  |  |  | HSP90 | | MIF | | MT20 | | MyticinB | |
| Park | Site | Year | Median | Range | Median | Range | Median | Range | Median | Range |
| Katmai | Takli | 2015 | 11.88 | 9.31-14.96 | 18.41 | 12.63-20.75 | 12.02 | 10.93-20.73 | 8.97 | 6.86-17.06 |
|  |  | 2016 | 15.58 | 11.01-17.07 | 18.76 | 13.66-21.19 | 11.90 | 9.10-15.14 | 11.29 | 5.80-17.96 |
|  | Kukak | 2015 | 14.28 | 9.66-15.27 | 18.24 | 12.71-22.95 | 10.49 | 9.09-12.15 | 12.73 | 6.76-16.99 |
|  |  | 2016 | 13.47 | 11.72-16.18 | 16.16 | 13.79-20.15 | 9.13 | 7.64-11.06 | 15.72 | 8.69-18.84 |
|  | Kaflia | 2015 | 15.84 | 12.49-17.89 | 18.09 | 13.32-21.03 | 10.27 | 9.15-11.16 | 15.80 | 5.58-19.32 |
|  |  | 2016 | 13.54 | 12.33-15.98 | 17.67 | 15.55-22.17 | 9.18 | 7.27-15.22 | 15.61 | 9.90-17.89 |
| Lake Clark | Fossil Point | 2015 | 13.87 | 11.34-16.36 | 18.25 | 14.39-20.12 | 11.23 | 10.59-13.74 | 8.60 | 6.76-15.74 |
|  |  | 2016 | 13.12 | 10.21-15.57 | 17.21 | 12.23-19.24 | 7.22 | 5.06-11.91 | 12.51 | 4.67-15.98 |
|  | Silver Salmon | 2015 | 12.60 | 10.75-15.19 | 17.24 | 12.37-21.61 | 10.78 | 8.71-18.94 | 11.62 | 7.80-17.60 |
|  |  | 2016 | 13.20 | 10.14-14.78 | 18.44 | 15.11-22.46 | 7.51 | 5.58-10.23 | 15.34 | 9.43-17.27 |
|  | Chinitna Bay | 2015 | 12.05 | 10.77-16.26 | 18.62 | 13.86-23.22 | 11.93 | 8.69-34.00 | 10.68 | 7.17-18.69 |
|  |  | 2016 | 14.06 | 10.20-15.21 | 16.20 | 11.92-20.42 | 6.59 | 4.43-8.18 | 11.61 | 6.70-19.72 |
|  | | | | | | | | | | |
|  |  |  | Mytilin | | P53 | |  |  |  |  |
| Park | Site | Year | Median | Range | Median | Range |  |  |  |  |
| Katmai | Takli | 2015 | 16.17 | 12.61-17.86 | 13.08 | 10.31-17.78 |  |  |  |  |
|  |  | 2016 | 15.27 | 12.35-19.13 | 12.50 | 11.73-14.78 |  |  |  |  |
|  | Kukak | 2015 | 15.04 | 11.86-17.45 | 12.16 | 9.39-34.00 |  |  |  |  |
|  |  | 2016 | 18.00 | 15.63-19.09 | 13.70 | 11.72-15.28 |  |  |  |  |
|  | Kaflia | 2015 | 16.87 | 12.89-21.17 | 12.12 | 11.37-13.27 |  |  |  |  |
|  |  | 2016 | 17.25 | 14.91-22.11 | 14.13 | 12.71-15.13 |  |  |  |  |
| Lake Clark | Fossil Point | 2015 | 15.67 | 12.02-17.25 | 12.04 | 11.16-14.57 |  |  |  |  |
|  |  | 2016 | 13.26 | 10.69-15.71 | 12.84 | 11.52-13.83 |  |  |  |  |
|  | Silver Salmon | 2015 | 15.87 | 13.45-16.61 | 12.23 | 10.02-14.43 |  |  |  |  |
|  |  | 2016 | 15.86 | 14.55-18.00 | 13.34 | 11.67-15.73 |  |  |  |  |
|  | Chinitna Bay | 2015 | 17.28 | 13.97-34.00 | 12.66 | 11.43-34.00 |  |  |  |  |
|  |  | 2016 | 14.98 | 12.15-19.24 | 12.68 | 11.33-14.66 |  |  |  |  |
